# An Analytic Approach for Optimal Geometrical Design of GaAs Nanowires for Maximal Light Harvesting in Photovoltaic Cells

**DOI:** 10.1038/srep46504

**Published:** 2017-04-20

**Authors:** Dan Wu, Xiaohong Tang, Kai Wang, Xianqiang Li

**Affiliations:** 1 OPTIMUS, Centre for OptoElectronics and Biophotonics, School of Electrical and Electronic Engineering, Nanyang Tech-nological University, 50 Nanyang Avenue, 639798, Singapore; 2 Department of Electrical & Electronic Engineering, South University of Science and Technology of China, 1088 Xueyuan Avenue, Shenzhen, 518055, China

## Abstract

Semiconductor nanowires(NWs) with subwavelength scale diameters have demonstrated superior light trapping features, which unravel a new pathway for low cost and high efficiency future generation solar cells. Unlike other published work, a fully analytic design is for the first time proposed for optimal geometrical parameters of vertically-aligned GaAs NW arrays for maximal energy harvesting. Using photocurrent density as the light absorbing evaluation standard, 2 μm length NW arrays whose multiple diameters and periodicity are quantitatively identified achieving the maximal value of 29.88 mA/cm^2^ under solar illumination. It also turns out that our method has wide suitability for single, double and four different diameters of NW arrays for highest photon energy harvesting. To validate this analytical method, intensive numerical three-dimensional finite-difference time-domain simulations of the NWs’ light harvesting are also carried out. Compared with the simulation results, the predicted maximal photocurrent densities lie within 1.5% tolerance for all cases. Along with the high accuracy, through directly disclosing the exact geometrical dimensions of NW arrays, this method provides an effective and efficient route for high performance photovoltaic design.

To meet the globally huge energy demanding problem and to cut the carbon pollution that causes climate change and threatens public health, nowadays photovoltaic technology has received unprecedented attention and historic investment world-wide. Despite the great effort devoted, for large scale implementation, price reduction is still the main concern to enable the solar cells to be fully cost-competitive with traditional energy sources. In recent years, III-V semiconductor nanowires(NWs) are attracting intense interest as a promising candidate in cost-efficient photovoltaic application due to their unique optical and electrical properties to greatly enhance the performance of devices^1^,^2^. It has been reported that the highest power conversion efficiency of GaAs NWs solar cells reached 15.3% in 2016^3^. On the optical side, theoretical analysis and experimental measurements demonstrate that single NW in planar manner can support leaky mode resonance(LMR) which enables the NW to concentrate and absorb light efficiently^4^,^5^. Well-arranged and vertically-aligned NW arrays also exhibit enhanced absorption and reduced reflectivity as compared with planar film-based devices^6^,^7^. On the electrical side, the high aspect ratio of the NWs decouples light absorption and carrier collection into orthogonal spatial directions^8^. Along the axis of NWs, effective light trapping can harvest large portion of incident photon energy whereas along the radial direction of NWs, the short carrier collection length significantly improves diffusion and collection efficiencies. There are also other fascinating features for NWs based photovoltaic devices^9^,^10^ including a variety of available methods to grow and tune the wide range of dimensions of NWs^11^, relaxation of the lattice-matching constraint through radial strain relaxation^12^,^13^,^14^,^15^, and the ability to integrate on low cost substrate^11^,^16^. Among various semiconductor materials, GaAs is drawing great attention due to the potential to achieve excellent device performance since they hold high absorption coefficient, ideal band gap and are capable of incorporation core-shell or axial multi-junction structures^17^,^18^.

Considering the above superior properties, tremendous efforts have been devoted to geometrical design of NWs to maximize light harvesting capability. Geometrical parameters such as diameter of NWs, periodicity, length and arrangement are numerically studied about their effect on photon energy absorption. In general, tuning the diameter of NWs results in the change of the resonant wavelengths within the NWs^19^ and through finite-difference time-domain(FDTD) simulation a near-unity broadband light absorption can be achieved^20^. Periodicity plays an important role in affecting the coupling among adjacent NWs. Therefore, it controls the turning point from LMR dominating in individual NWs to the photonic Bloch modes governing the coupled NW arrays^21^,^22^. Martin Foldyna *et al*. compared squarely and hexagonal periodic NW arrays and confirmed that, regardless of the arrangement, volume filling ratio mattered greatly to the light confinement^23^. Despite the efforts mentioned above, decision on the optimal arrangement of NW arrays is still a challenging issue since the optical processes involved are interrelated and highly dispersive. They include the diameter-dependent resonant wavelength selectivity, material dispersive properties across the incident solar spectrum as well as reflectance and transmittance minimization to maximize the absorption of NWs. As a result, this multi-parameters optimization problem is mostly solved by fully numerical simulations of given parameter spaces for different publications. Consequently, the obtained optimal values are parameter-space-determinant let alone the time-consuming process. To solve this problem, Sturmberg *et al*. proposed a semi-analytic method to rationally narrow down the optimal geometrical parameters of a given NW arrays in certain range which was applicable to various materials^24^. Nevertheless, the confined range is still among hundreds of nanometers for periodicity and is limited to single diameter NW arrays. Moreover, although the number of numerical simulations has been noticeably reduced, they should also be accompanied to achieve the optimal design. Hence, an effective and efficient fully analytic design is greatly desired to directly find out the optimal geometrical parameters for maximal light absorption regardless of single or multiple diameters NW arrays for photovoltaic application.

In this paper, we propose a fully analytic method to calculate the optimal geometrical parameters for GaAs NW arrays in order to gain the highest photon harvesting. Diameters of the NWs are computed based on the LMR and Mie resonant theory. The optimal periodicity of the NW arrays is determined according to the equivalent thin film method to minimize light reflection and transmission. Moreover, single, double, and four diameters of NW arrays are discussed to obtain each optimized geometrical parameters. Intensive numerical simulations are also carried out to verify the presented method. The well matching of the largest photocurrent densities generated from the NW arrays with the calculated geometrical parameters and the values obtained from FDTD simulation results prove the effectiveness of the proposed method to guide the practical NWs based photovoltaic cells design.

## Results and Discussion

**Figure 1:**
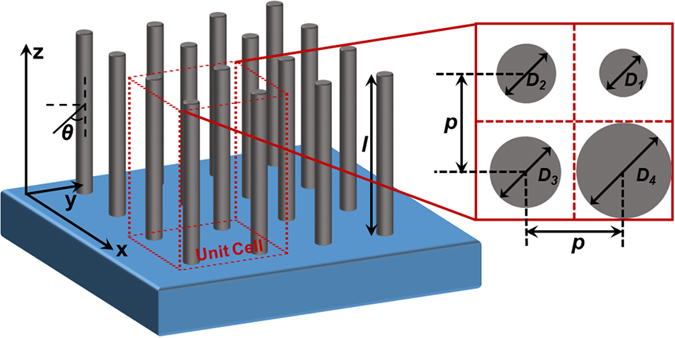
Schematic of vertically-aligned cylindrical nanowires(NWs) array of length *l* standing on a substrate and an enlarged unit cell showing characteristic dimensions of NW arrays including diameter *D*_*i*_ and periodicity *p*.

Vertically aligned GaAs NW arrays for our study is schematically shown in Fig. 1. This structure is in line with most of the semiconductor NWs based core-shell or hybrid solar cell configuration^25^,^26^. The NWs are squarely arranged with the smallest repeatable unit cells as identified in the Fig. 1 inset. The unit cell contains four NWs with the same or different diameters, *D*_*i*_. In this work, our analytic method will be applied in the single, double and four different diameters of NW arrays. In the single diameter case, all four diameters of NWs in the unit cell have the same value. In the double diameters case, the diameters of the diagonal NWs such as *D*_*1*_ and *D*_*3*_ or *D*_*2*_ and *D*_*4*_ have the same value whereas the NWs in the same columns and rows have different values. Owing to the symmetry of the unit cell, either pair of the same diameters of NWs can be named as primary NWs with their diameters as *D*_*prim*_ and the remaining two NWs are named as secondary NWs with their diameters as *D*_*second*_. Similarly, all diameters of the NWs have different values in the unit cell in the case of four diameters of NWs. The NWs are named according to the sequence in our analytic method to obtain their diameters and are therefore named as primary, secondary, third and fourth NWs. Besides, the same center to center distance between the adjacent NWs is named as periodicity, *p*. The volume filling ratio(FR) for the NWs of the cell is defined as *π(D*_*1*_^*2*^ + *D*_*2*_^*2*^ + *D*_*3*_^*2*^ + *D*_*4*_^*2*^)*/16p*^*2*^ which has a maximum value of *π/4* when the NWs take up the maximum volume percentage of an unit cell^27^. All of the NWs have the same length, *l* and it is reported that the absorption of the NW arrays increase with the length of NWs logarithmically^23^. However, considering the fabrication difficulty, NWs length of 2 μm is chosen for the following discussion.

**Figure 2:**
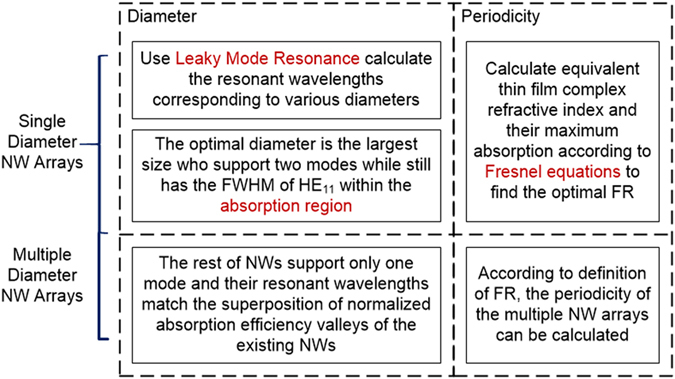
Flowchart of analytically geometrical dimension design for single, double and four different diameters of NW arrays.

To quantitatively determine each geometrical parameter of NW arrays, the interrelated and highly dispersive light trapping processes should be separately considered. In general, there are two dominant processes that govern the light absorption including diameter-determinant mode resonance within the NWs and the periodicity-affected reflection and transmission of incident light. The remaining issue is to construct the relationships among the geometrical parameters and each light absorbing process to guarantee maximal light absorption. A flowchart of the presented analytic design is shown in Fig. 2 and four diameters of NW arrays are illustrated as the design example. Geometrical parameters of single and double diameters of NW arrays as the simpler cases can also be obtained during the process. During the calculation of the diameter of NWs, the assumption is taken as no coupling among NWs since the coupling among NWs will cause the incident photon energy competence which will decrease the light absorption. To determine the value of the diameter of the primary NW, the LMR is used to obtain the minimum and maximum diameter values that support at least two modes(HE_11_ and HE_12_) which fall in the absorbing region^4^. The absorbing region is defined as the superposition of incident AM 1.5 G spectrum^28^ and the material absorbing range. At the same time, the normalized absorption efficiency of each NW in the obtained diameter range is calculated from the Mie theory^5^. Upon acquisition of the optimal value of the primary diameter, the secondary NW is found to satisfy two conditions: 1) only one mode should be excited within the secondary NWs; 2) the resonant wavelength of the secondary NWs should match the valley of the primary diameter’s normalized absorption efficiency spectrum^29^,^30^. Similarly, the third and fourth diameter of NWs are calculated to satisfy the requirements that each of them should support only one resonant mode and their respective resonant wavelengths should match the valleys of the superposition of normalized absorption efficiency spectrum of the primary and secondary NWs’. In this way, all four diameters are rationally determined. Furthermore, periodicity of NW arrays is solved by equivalent thin film method which can represent the reflection and transmission of the NW arrays under solar illumination^31^,^32^. Since this equivalent relationship is built on the basis of the volume ratio of NWs material, it has nothing to do with the diameters variation as long as FR is fixed. Consequently, once the optimal FR is obtained, it is suitable for various diameter combinations of NW arrays. In the end, along with the optimal diameter values, the periodicities can be calculated from the definition of FR and all of the geometrical dimensions are acquired.

At this stage, the optimal diameters and the FR are determined and the corresponding periodicities can be acquired from the definition of the FR. With the optimal geometrical dimensions, the NW arrays should lead to maximal light absorption. Photocurrent density *J*_*ph*_ is mostly used to measure the light harvesting capability assuming that every absorbed photon leads to an exciton separation followed by a successful carrier collection. The definition is 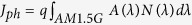 where *A(λ*) is the absorptance inside nanowires as a function of the incident wavelength, *N(λ*) is the number of photons per unit area per second for the incident wavelength from the standard solar spectrum. At the same time, FDTD numerical simulation(Lumerical FDTD Solutions 8.15) is adopted to compare with our proposed method.

### Analytic design for single diameter GaAs NW arrays

**Figure 3:**
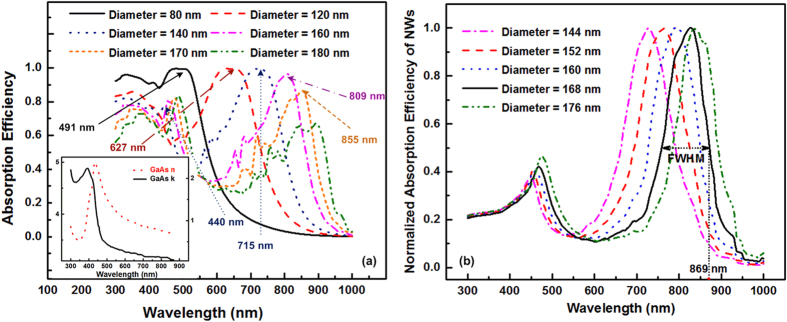
(**a**) Absorption efficiency of GaAs NW arrays change with incident wavelength with FR of 0.05 and the insets illustrate the complex refractive index for GaAs;(**b**) normalized absorption efficiency of GaAs NWs calculated by Mie theory.

As shown in Fig. 3, the absorption efficiency of the GaAs NW arrays is obtained from FDTD simulation with FR of 0.05. The refractive index and the extinction coefficients of the respective materials are supplemented in Fig. 3(a) inset. The band gap of GaAs is 1.43 eV^33^ corresponding to critical wavelength of 869 nm. Figure 3(a) also illustrates the red-shifted evolvement of the HE_11_ resonant wavelengths with the increase of diameter of NWs. Using the dispersion relation, we calculate the resonant wavelengths of HE_11_ mode related to each diameter from 50 to 200 nm for GaAs NWs by taking the limit of propagation constant along the axis of NWs as zero. Although in the situation that wavevector aligned perfectly parallel to NWs axis, the eigenvalue equation is ill-defined, the wave front of the free space plane wave will be perturbed by the high index of NW arrays introducing transverse components to the wavevector which allows the incident light to couple into the leaky modes. This guarantees the use of limit propagation constant to be zero^4^,^21^,^24^. The calculated resonant wavelengths are marked on respective peaks. The well agreements of the calculated resonant peaks and those obtained from FDTD simulation demonstrate the effectiveness of our method. On one hand, both calculated and simulation results show that diameter of 140 nm is the turning point from one mode to two modes resonance within GaAs NWs. On the other hand, the resonant peak of 869 nm corresponding to the diameter of 174 nm is the upper limit of the absorbing region for GaAs material. The normalized absorption efficiency for GaAs NWs where two modes existed is calculated by Mie theory as shown in Fig. 3(b). It clearly displays that the maximum diameters for GaAs is 168 nm while keeping FWHM lying within each absorption region. Compared with our method, the experimentally reported highest efficiency GaAs NW arrays solar cell whose optimized diameter of NWs is 165 nm^34^. The narrow difference of 3 nm in diameter prediction proves the effectiveness of this method.

**Figure 4:**
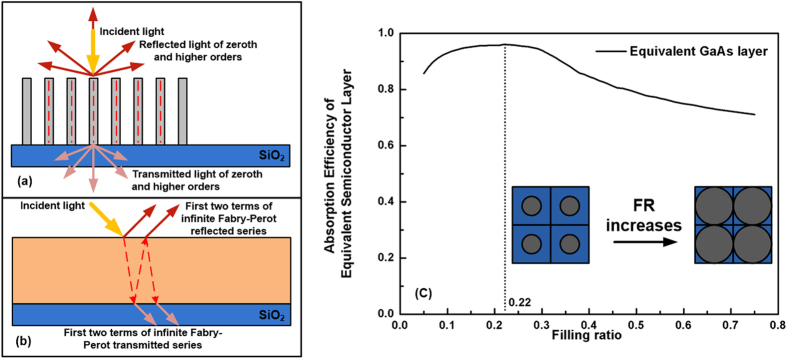
(**a,b**) are the schematics showing equivalent thin film method whereas(**c**) is absorption efficiency of equivalent thin films for GaAs NW arrays as a function of FR.

The FRs for GaAs is optimized according to equivalent thin film method as schematically shown in Fig. 4(a,b). An artificial thin film is created to represent the reflection and transmission behavior of the NW arrays. The diameter of NWs and periodicity is therefore removed and a mesoporous equivalent thin film is built with the thickness of the length of NWs. Detailed mathematics derivations can be found in the supporting information of the reference^24^. Figure 4(c) shows that the absorption of the equivalent layers for GaAs materials rise initially with the increase of FR and then fall down after reach their optimal value. The optimal FR values correspond to the ones that maximize the light absorption of the equivalent layer when considering the Fresnel reflection and transmission. The trend is mainly due to the variation of the transmission and reflection of the equivalently complex refractive index due to FR change. When FR grows from 0.05 to the optimal value, the buildup of the III-V material increase light absorption and reduce transmission. However, too large portion of NWs in the unit cell will increase the reflection of the incident light which have also been confirmed in the publication^24^. Thus, the optimal FR for GaAs NWs is 0.22. According to the definition of FR, we can calculate the optimal periodicity of the single diameter GaAs NWs as 317.43 nm.

**Figure 5:**
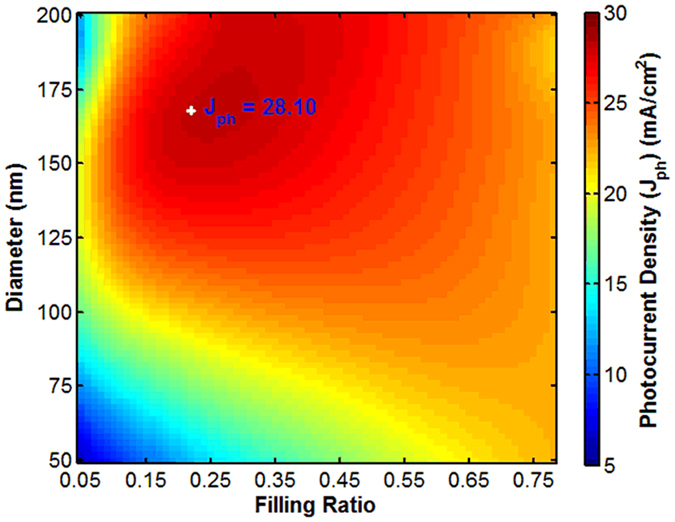
Theoretical predicted maximum value compared with FDTD simulation for single diameter GaAs NW arrays.

Photocurrent densities for single diameter design are shown in Fig. 5 from FDTD simulations and the optimal values obtained from the proposed method marked in bold. The maximal value for the *J*_*ph*_ obtained from the FDTD is 28.18 mA/cm^2^ whereas the maximum *J*_*ph*_ calculated from our proposed method is 28.10 mA/cm^2^. The calculation error for GaAs NWs is 0.28%. This confirms that our proposed method is very effective in predicting the maximum light absorption for single diameter NW arrays.

### Analytic design for double diameters GaAs NW arrays

Adding another diameter of NWs in the unit cell has been investigated by many researchers with the purpose to further boost light absorption efficiency^20^,^23^ since adding a secondary diameter NWs can supplement the selectively absorption spectrum due to the resonant effect and thus improve energy harvesting capability. However, the secondary diameter are usually chosen and validated by intensive numerical simulations. From the above discussion, the secondary diameter should be thin enough to reduce reflection but still is required to support one mode. At the same time, the absorption valley of primary NWs should be complemented by the resonant wavelength of the second NWs. As a result, from normalized absorption efficiency of primary diameter of 168 nm GaAs NWs calculated by the Mie theory in Fig. 3(b), the absorption valleys are found to locate at 600 nm. Therefore, diameter of 114 nm GaAs NWs satisfies the selection criteria of the secondary diameter. At the same time, the FRs calculation method is still valid leading to 0.22 for GaAs NWs. According to definition of FR, periodicities are calculated to be 270 nm for GaAs NWs.

**Figure 6:**
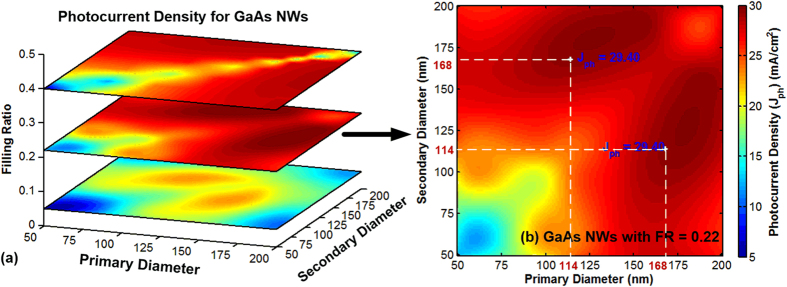
Overview (**a**) and extracted slice for FR is 0.22(**b**) of theoretical predicted maximum value compared with FDTD simulation for double diameter GaAs NW arrays.

As shown in Fig. 6, photocurrent densities vary with different primary and secondary diameter combinations as well as FRs for GaAs NWs. Figure 6(a) gives the overall view of the three parameters changing whereas Fig. 6(b) extracts the slice of the optimal FRs for GaAs NWs. FR demonstrates similar trend that predicted by equivalent thin film method. The photocurrent densities generally increase with FRs, rise to its maximum value and then fall down. Although only three FRs are displayed for each material, they are deliberately chosen to gain a whole picture of the FR’s influence according to Fig. 4(c). Obviously, large values of photocurrent densities have been achieved with optimal FRs for GaAs NWs. We marked the photocurrent densities on the Fig. 6(b) for our purposed method as 29.4 mA/cm^2^ for GaAs NW arrays respectively. The symmetrical distribution of the photocurrent density coincides with the symmetrical distribution of two diameters NWs in the unit cell. To estimate the tolerance, the maximal photocurrent densities obtained from FDTD is 29.56 mA/cm^2^ for GaAs NW arrays, which leads to a tolerance of 0.5%. The light absorption spectrum from numerical simulation of the optimal two diameter NW arrays is provided in Fig. SI1 demonstrating the well combinations of NWs for high light absorption.

### Analytic design for four diameters GaAs NW arrays

In the case of four diameters of NWs, all diameters of the NWs have different values in the unit cell. It is reported that higher absorption efficiency is obtained when multiple diameters are involved in the unit cell^20^ compared with single or double diameters NW arrays. However, due to the numerous simulation cases needed, validation of multi-diameter NWs across parameter spaces of reasonable number of diameters of NWs combinations has seldom been reported because of the large amount of time needed for data acquisition. In this section, we will demonstrate the effectiveness of our proposed method in predicting the largest photon energy absorption by directly providing the geometrical parameters of NW arrays. FDTD simulations are also accompanied to confirm the feasibility of our method.

Similarly with double diameter NWs case, the selection of the third and the fourth diameters of NWs are chosen to match the absorption valley of the superposition of the primary and secondary NWs’ absorption efficiency calculated by the Mie theory. Upon acquisition of the superposition of the normalized absorption efficiency of the optimal primary and secondary diameters for GaAs NWs, the absorption valleys can be easily witnessed as 523 and 720 nm for GaAs. Therefore, the diameter of NWs whose resonant wavelength matches the absorption valley of respect curves are chosen as 92 and 140 nm for GaAs NWs.

**Figure 7:**
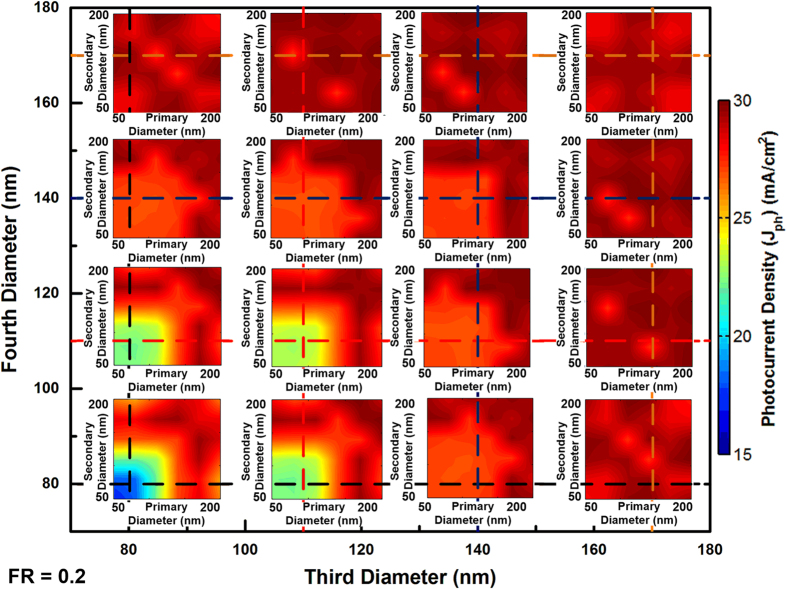
Theoretical predicted maximum value compared with FDTD simulation for four diameter GaAs NW arrays.

Since all of the four diameters of NWs are determined, according to the optimal FR shown in Fig. 4(c) and the relationship of FR and periodicity, the periodicities is calculated to be 248.67 nm. At the same time, FDTD simulations run across each diameter parameter space are provided. Considering there are four different diameters to be independent variables, it is difficult to have a direct view of the result in conventional two or three dimensional figures. Enumeration method is employed to demonstrate simulation results as shown in Fig. 7. There are two sets of coordinate systems say, inner and outer. The *x* and *y* axes of the inner coordinate system represent the primary and secondary diameter NWs. The *x* and *y* axes of the outer coordinate system denote the third and fourth diameter NWs. Each intersect of the dash lines indicates different combination of the third and fourth diameters whereas the primary and secondary diameter run through 50 to 200 nm. Due to the limited length of the paper, representative values are chosen in the way to have a full picture of the result. For GaAs NW arrays, diameter of 80 nm denotes single mode existence within NWs and 110 nm is selected to show the transition from single mode to double mode. 140 nm represent the starting of the double mode within NWs and 170 nm demonstrates the absorbing edge of the NWs. The photocurrent densities for our proposed calculation method are obtained as 29.88 mA/cm^2^ compared with simulation results, the tolerance is 1.5%. The absorption spectrum of optimal four diameter NW arrays from FDTD simulations is provided in Fig. SI2 and near-unity absorption can be achieved across the incident spectrum.

## Conclusions

In this paper, a fully analytic design method is proposed to calculate the geometrical dimensions of NW arrays for maximal light absorption for photovoltaic applications. Specifically, the diameters of NWs and periodicity are rationally determined with low tolerance and within a short period of time as compared to conventional three dimensional full FDTD simulations. Single, double and four diameters of GaAs NW arrays are designed to pursue the largest photocurrent densities. Considering 2 μm length NW arrays, the primary diameters of 168 nm for GaAs NWs are obtained both from LMR and Mie theory to excite two resonant modes while remain their FWHM of HE_11_ mode in the absorbing region. By matching the absorption valley of the primary diameter, the optimal diameters for the secondary GaAs NWs are 114 nm. Moreover, broadband high absorption is achieved for four diameters NW arrays through matching their respective resonant wavelengths and the valleys of the superposition of the primary and secondary NWs’ normalized absorption efficiency spectrum. Therefore, the third and fourth diameters are 92 and 140 nm for GaAs NWs. At the same time, the equivalent thin film method combined with Fresnel reflection and transmission lead to the optimal FR of 0.22 for GaAs NWs despite various diameters combinations. The optimal geometrical dimensions of four diameters NW arrays lead to the highest photocurrent density of 29.88 mA/cm^2^ for GaAs respectively. Moreover, the validity of the proposed method is verified through intensive numerical simulations across the same parameter spaces. The precision of the prediction of maximal light absorption compared with simulation results lies within 1.5% for all NWs cases. The wide suitability, high precision and time saving capability in predicting the geometrical dimensions of NW arrays for maximal light absorption demonstrate the effectiveness of the proposed method in design of future efficient solar cell.

## Methods

### Theoretical simulations

FDTD numerical simulation is adopted to compare with our proposed method. The boundary condition in the simulation is set as periodic boundary condition along *x* and *y* directions whereas perfect matching layer is applied along the z axis as shown in Fig. 1. The NW arrays of given geometry and arrangement are vertically standing on SiO_2_ substrate using Palik material data provided by Lumerical. The diameter of NWs ranges from 50 to 200 nm and FR falls in the range of 0.05 to π/4. Broadband(350–1000 nm), non-polarized, and infinite plane wave source is incident upon the NWs whose direction is perpendicular to the SiO_2_ substrate.

## Additional Information

**How to cite this article:** Wu, D. *et al*. An Analytic Approach for Optimal Geometrical Design of GaAs Nanowires for Maximal Light Harvesting in Photovoltaic Cells. *Sci. Rep.* **7**, 46504; doi: 10.1038/srep46504(2017).

**Publisher's note:** Springer Nature remains neutral with regard to jurisdictional claims in published maps and institutional affiliations.

## Supplementary Material

Supplementary Information
