# Supplementary material for: An Analytic Approach for Optimal Geometrical Design of GaAs Nanowires for Maximal Light Harvesting in Photovoltaic Cells
Source: Sci Rep. 2017 Apr 20;7:46504. doi: 10.1038/srep46504 (PMC5397838; doi:10.1038/srep46504)
Supplement: Supplementary Information [file srep46504-s1.pdf]

An Analytic Approach for Optimal Geometrical Design of GaAs Nanowires for Maximal Light Harvesting in Photovoltaic Cells

——Supplementary Information

Dan Wu [1]*, Xiaohong Tang [1], Kai Wang [2], Xianqiang Li [1]

[1] *OPTIMUS, Centre for OptoElectronics and Biophotonics, School of Electrical and Electronic Engineering, Nanyang Tech-nological University, 50 Nanyang Avenue, 639798, SINGAPORE*

[2] *Department of Electrical & Electronic Engineering, South University of Science and Technology of China, 1088 Xueyuan Avenue, Shenzhen, 518055, CHINA*

1. Light absorption of individual NWs and the whole arrays in multi-diameter NW arrays

For double diameter NW arrays with optimal geometrical dimensions and arrangement, the light absorption of individual NWs and the whole arrays is shown in the Fig. SI1 which demonstrates the well combinations of NWs for high light absorption.

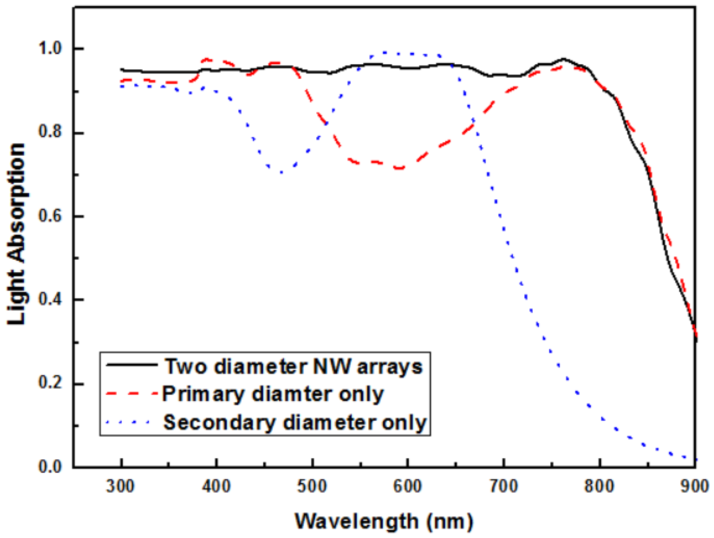

Figure SI1. Absorption spectrum wavelength for optimal two diameter NW arrays including each individual NW in the optimized diameters.

For four diameter NW arrays with optimal geometrical dimensions and arrangement, the light absorption of individual NWs and the whole array is shown in the Fig. SI2.

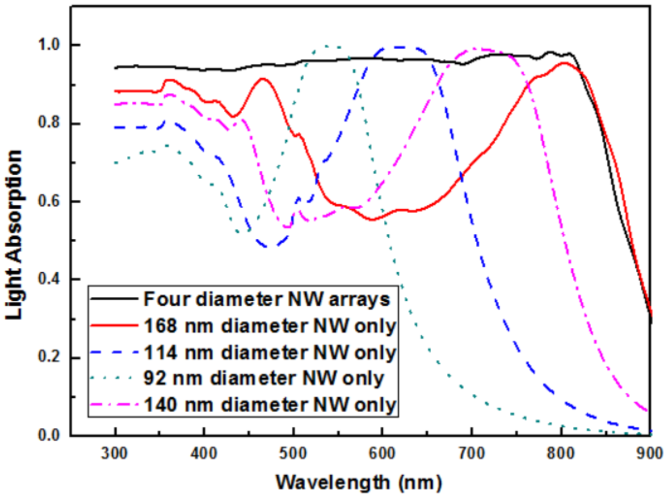

Figure SI2. Absorption spectrum wavelength for optimal four diameter NW arrays including each individual NW in the optimized diameters.
